# Supplementary material for: Meta-Analysis and Gene Set Enrichment Relative to ER Status Reveal Elevated Activity of MYC and E2F in the “Basal” Breast Cancer Subgroup
Source: PLoS One. 2009 Mar 9;4(3):e4710. doi: 10.1371/journal.pone.0004710 (PMC2650420; doi:10.1371/journal.pone.0004710)
Supplement: Dataset S1 — GSEA results from first screen. The file contains a zipped archive of the HTML results of the second GSEA screen. Reports for enriched or depleted datasets may be accessed from the “index.html” link within the my_analysis.GseaPreranked.1219975950625.rpt.zip ZIP archive. Enrichment plots are available for each of the 50 most enriched and depleted gene sets. (13.32 MB ZIP) [file pone.0004710.s001.zip › my_analysis.GseaPreranked.1219975950625.rpt.zip!//BRCA_BRCA1_NEG.html]

Details for gene set BRCA\_BRCA1\_NEG[GSEA]

|  || Dataset | zSco.ERneg.to.ERpos\_collapsed |
| Phenotype | NoPhenotypeAvailable |
| Upregulated in class | na\_neg |
| GeneSet | BRCA\_BRCA1\_NEG |
| Enrichment Score (ES) | -0.698383 |
| Normalized Enrichment Score (NES) | -3.402925 |
| Nominal p-value | 0.0 |
| FDR q-value | 0.0 |
| FWER p-Value | 0.0 |
Table: GSEA Results Summary

  

Fig 1: Enrichment plot: BRCA\_BRCA1\_NEG      
 Profile of the Running ES Score & Positions of GeneSet Members on the Rank Ordered List

  

| PROBE | GENE SYMBOL | GENE\_TITLE | RANK IN GENE LIST | RANK METRIC SCORE | RUNNING ES | CORE ENRICHMENT || 1 | PRRX2 |  |  | 341 | 4.825 | -0.0116 | No |
| 2 | TXNDC13 |  |  | 932 | 3.581 | -0.0458 | No |
| 3 | EPHA4 |  |  | 2467 | 2.218 | -0.1556 | No |
| 4 | USP22 |  |  | 2924 | 1.973 | -0.1843 | No |
| 5 | HEY1 |  |  | 3698 | 1.621 | -0.2382 | No |
| 6 | CDC42BPA |  |  | 4330 | 1.379 | -0.2820 | No |
| 7 | DNAJA4 |  |  | 4395 | 1.357 | -0.2828 | No |
| 8 | PARVA |  |  | 4396 | 1.357 | -0.2788 | No |
| 9 | PALMD |  |  | 4722 | 1.246 | -0.2998 | No |
| 10 | TFPI |  |  | 5091 | 1.119 | -0.3244 | No |
| 11 | BMPR2 |  |  | 5565 | 0.954 | -0.3574 | No |
| 12 | KLF9 |  |  | 5597 | 0.945 | -0.3570 | No |
| 13 | NPTN |  |  | 5846 | 0.861 | -0.3733 | No |
| 14 | BRD8 |  |  | 6010 | 0.808 | -0.3832 | No |
| 15 | SPAG5 |  |  | 6030 | 0.800 | -0.3823 | No |
| 16 | HDGFRP3 |  |  | 6446 | 0.668 | -0.4118 | No |
| 17 | C5ORF13 |  |  | 6512 | 0.645 | -0.4148 | No |
| 18 | DKFZP686A01247 |  |  | 6610 | 0.618 | -0.4204 | No |
| 19 | PLCXD1 |  |  | 6655 | 0.606 | -0.4219 | No |
| 20 | PDLIM3 |  |  | 6847 | 0.535 | -0.4348 | No |
| 21 | ETFB |  |  | 6985 | 0.493 | -0.4438 | No |
| 22 | MATN2 |  |  | 7154 | 0.435 | -0.4552 | No |
| 23 | TSPAN8 |  |  | 7315 | 0.378 | -0.4662 | No |
| 24 | PNMT |  |  | 7325 | 0.374 | -0.4658 | No |
| 25 | SPDEF |  |  | 7330 | 0.372 | -0.4650 | No |
| 26 | SLC9A2 |  |  | 7351 | 0.367 | -0.4654 | No |
| 27 | RMND5B |  |  | 7446 | 0.330 | -0.4716 | No |
| 28 | ANKRD25 |  |  | 7470 | 0.321 | -0.4724 | No |
| 29 | ASTN2 |  |  | 7528 | 0.296 | -0.4758 | No |
| 30 | PRKD1 |  |  | 7686 | 0.236 | -0.4871 | No |
| 31 | AFF1 |  |  | 7753 | 0.212 | -0.4914 | No |
| 32 | SNPH |  |  | 7912 | 0.142 | -0.5030 | No |
| 33 | INPP4B |  |  | 8044 | 0.088 | -0.5127 | No |
| 34 | MPDZ |  |  | 8133 | 0.062 | -0.5192 | No |
| 35 | SPON1 |  |  | 8298 | 0.002 | -0.5316 | No |
| 36 | RAB3B |  |  | 8594 | -0.126 | -0.5536 | No |
| 37 | WT1 |  |  | 8652 | -0.153 | -0.5575 | No |
| 38 | CRYM |  |  | 8659 | -0.155 | -0.5575 | No |
| 39 | PRKG1 |  |  | 8683 | -0.162 | -0.5588 | No |
| 40 | TAOK1 |  |  | 8845 | -0.229 | -0.5703 | No |
| 41 | PALLD |  |  | 8851 | -0.232 | -0.5700 | No |
| 42 | DOPEY2 |  |  | 8862 | -0.238 | -0.5700 | No |
| 43 | GPR98 |  |  | 8941 | -0.282 | -0.5751 | No |
| 44 | CHGA |  |  | 9061 | -0.342 | -0.5831 | No |
| 45 | ITGB6 |  |  | 9148 | -0.380 | -0.5885 | No |
| 46 | KIAA0100 |  |  | 9323 | -0.470 | -0.6003 | No |
| 47 | ENC1 |  |  | 9363 | -0.491 | -0.6019 | No |
| 48 | GREM1 |  |  | 9466 | -0.546 | -0.6080 | No |
| 49 | TRAF4 |  |  | 9477 | -0.552 | -0.6071 | No |
| 50 | NEO1 |  |  | 9529 | -0.577 | -0.6093 | No |
| 51 | DUSP13 |  |  | 10045 | -0.874 | -0.6457 | No |
| 52 | SERPINF1 |  |  | 10347 | -1.059 | -0.6655 | No |
| 53 | BEXL1 |  |  | 10353 | -1.064 | -0.6627 | No |
| 54 | DUSP6 |  |  | 10680 | -1.305 | -0.6836 | No |
| 55 | WWOX |  |  | 10694 | -1.313 | -0.6807 | No |
| 56 | GOSR1 |  |  | 10837 | -1.419 | -0.6872 | No |
| 57 | TSPYL4 |  |  | 10985 | -1.555 | -0.6938 | Yes |
| 58 | SC5DL |  |  | 10994 | -1.562 | -0.6898 | Yes |
| 59 | IGFBP7 |  |  | 11014 | -1.577 | -0.6865 | Yes |
| 60 | MID1IP1 |  |  | 11122 | -1.682 | -0.6897 | Yes |
| 61 | DIP2C |  |  | 11206 | -1.747 | -0.6908 | Yes |
| 62 | FBXL2 |  |  | 11222 | -1.760 | -0.6867 | Yes |
| 63 | HSD11B2 |  |  | 11245 | -1.780 | -0.6831 | Yes |
| 64 | STXBP1 |  |  | 11435 | -1.972 | -0.6916 | Yes |
| 65 | SPARC |  |  | 11451 | -1.990 | -0.6869 | Yes |
| 66 | UNC119 |  |  | 11464 | -2.000 | -0.6819 | Yes |
| 67 | PLK2 |  |  | 11632 | -2.151 | -0.6882 | Yes |
| 68 | LASP1 |  |  | 11661 | -2.179 | -0.6839 | Yes |
| 69 | TSPAN15 |  |  | 11730 | -2.249 | -0.6824 | Yes |
| 70 | VEZF1 |  |  | 11771 | -2.295 | -0.6786 | Yes |
| 71 | AHNAK |  |  | 11844 | -2.364 | -0.6771 | Yes |
| 72 | SCAMP1 |  |  | 11847 | -2.367 | -0.6702 | Yes |
| 73 | NUAK1 |  |  | 11863 | -2.389 | -0.6643 | Yes |
| 74 | ZDHHC17 |  |  | 11868 | -2.399 | -0.6575 | Yes |
| 75 | GPC4 |  |  | 11878 | -2.414 | -0.6510 | Yes |
| 76 | GJA1 |  |  | 11883 | -2.421 | -0.6442 | Yes |
| 77 | CA2 |  |  | 11917 | -2.453 | -0.6394 | Yes |
| 78 | GPRC5C |  |  | 11948 | -2.493 | -0.6343 | Yes |
| 79 | COL3A1 |  |  | 11953 | -2.497 | -0.6272 | Yes |
| 80 | RPL23A |  |  | 11964 | -2.518 | -0.6205 | Yes |
| 81 | GPC1 |  |  | 11965 | -2.518 | -0.6131 | Yes |
| 82 | MAP3K1 |  |  | 11996 | -2.557 | -0.6078 | Yes |
| 83 | GOLPH2 |  |  | 12127 | -2.743 | -0.6096 | Yes |
| 84 | TP53AP1 |  |  | 12136 | -2.753 | -0.6020 | Yes |
| 85 | MYLIP |  |  | 12172 | -2.805 | -0.5964 | Yes |
| 86 | SERPINA6 |  |  | 12203 | -2.846 | -0.5902 | Yes |
| 87 | RETSAT |  |  | 12237 | -2.897 | -0.5842 | Yes |
| 88 | PDE8B |  |  | 12262 | -2.924 | -0.5773 | Yes |
| 89 | TNRC9 |  |  | 12280 | -2.941 | -0.5699 | Yes |
| 90 | SORD |  |  | 12298 | -2.964 | -0.5624 | Yes |
| 91 | MAST4 |  |  | 12400 | -3.100 | -0.5609 | Yes |
| 92 | RNF43 |  |  | 12408 | -3.113 | -0.5522 | Yes |
| 93 | MEIS3P1 |  |  | 12414 | -3.125 | -0.5434 | Yes |
| 94 | TEGT |  |  | 12420 | -3.138 | -0.5345 | Yes |
| 95 | CSNK1A1 |  |  | 12437 | -3.160 | -0.5263 | Yes |
| 96 | CDH11 |  |  | 12438 | -3.164 | -0.5170 | Yes |
| 97 | MATN3 |  |  | 12441 | -3.165 | -0.5077 | Yes |
| 98 | ATBF1 |  |  | 12447 | -3.174 | -0.4987 | Yes |
| 99 | NQO1 |  |  | 12453 | -3.182 | -0.4897 | Yes |
| 100 | SULT1A2 |  |  | 12505 | -3.268 | -0.4839 | Yes |
| 101 | CXCR7 |  |  | 12523 | -3.295 | -0.4754 | Yes |
| 102 | KIAA0644 |  |  | 12536 | -3.314 | -0.4665 | Yes |
| 103 | CLEC11A |  |  | 12595 | -3.422 | -0.4608 | Yes |
| 104 | ITGBL1 |  |  | 12600 | -3.440 | -0.4509 | Yes |
| 105 | SH3BP4 |  |  | 12671 | -3.558 | -0.4457 | Yes |
| 106 | SDF2 |  |  | 12687 | -3.589 | -0.4362 | Yes |
| 107 | CA12 |  |  | 12705 | -3.627 | -0.4268 | Yes |
| 108 | GALNT7 |  |  | 12708 | -3.629 | -0.4162 | Yes |
| 109 | CPE |  |  | 12769 | -3.790 | -0.4096 | Yes |
| 110 | HMGCS2 |  |  | 12771 | -3.794 | -0.3984 | Yes |
| 111 | CTSF |  |  | 12816 | -3.921 | -0.3901 | Yes |
| 112 | CGI-38 |  |  | 12835 | -3.967 | -0.3798 | Yes |
| 113 | PLA2G10 |  |  | 12843 | -3.988 | -0.3685 | Yes |
| 114 | IGFBP2 |  |  | 12897 | -4.147 | -0.3602 | Yes |
| 115 | ITGB5 |  |  | 12921 | -4.213 | -0.3495 | Yes |
| 116 | HAGH |  |  | 12925 | -4.250 | -0.3372 | Yes |
| 117 | TMEM16A |  |  | 12927 | -4.253 | -0.3247 | Yes |
| 118 | UQCRQ |  |  | 12939 | -4.284 | -0.3128 | Yes |
| 119 | C5ORF21 |  |  | 12941 | -4.293 | -0.3002 | Yes |
| 120 | STK39 |  |  | 12951 | -4.321 | -0.2881 | Yes |
| 121 | DUSP4 |  |  | 13001 | -4.456 | -0.2786 | Yes |
| 122 | FMOD |  |  | 13009 | -4.484 | -0.2659 | Yes |
| 123 | GATA3 |  |  | 13014 | -4.509 | -0.2528 | Yes |
| 124 | HTRA1 |  |  | 13042 | -4.623 | -0.2412 | Yes |
| 125 | PIB5PA |  |  | 13125 | -4.921 | -0.2329 | Yes |
| 126 | PLAT |  |  | 13127 | -4.926 | -0.2184 | Yes |
| 127 | CAMK2N1 |  |  | 13145 | -5.010 | -0.2048 | Yes |
| 128 | PPT1 |  |  | 13146 | -5.015 | -0.1900 | Yes |
| 129 | REEP1 |  |  | 13155 | -5.073 | -0.1756 | Yes |
| 130 | DCI |  |  | 13177 | -5.217 | -0.1618 | Yes |
| 131 | PH-4 |  |  | 13179 | -5.236 | -0.1463 | Yes |
| 132 | SYNJ2BP |  |  | 13207 | -5.444 | -0.1323 | Yes |
| 133 | GLI3 |  |  | 13213 | -5.527 | -0.1163 | Yes |
| 134 | ACOX2 |  |  | 13223 | -5.611 | -0.1004 | Yes |
| 135 | TBC1D9 |  |  | 13226 | -5.639 | -0.0838 | Yes |
| 136 | DIP13B |  |  | 13233 | -5.716 | -0.0674 | Yes |
| 137 | DNALI1 |  |  | 13258 | -6.058 | -0.0513 | Yes |
| 138 | PIGT |  |  | 13264 | -6.118 | -0.0335 | Yes |
| 139 | EMP2 |  |  | 13273 | -6.228 | -0.0157 | Yes |
| 140 | LRRC17 |  |  | 13294 | -6.494 | 0.0020 | Yes |
Table: GSEA details [plain text format]

  

Fig 2: BRCA\_BRCA1\_NEG: Random ES distribution      
 Gene set null distribution of ES for **BRCA\_BRCA1\_NEG**
